# Pathophysiological and Therapeutic Roles of Fascial Hyaluronan in Obesity-Related Myofascial Disease

**DOI:** 10.3390/ijms231911843

**Published:** 2022-10-06

**Authors:** Chiedozie Kenneth Ugwoke, Erika Cvetko, Nejc Umek

**Affiliations:** Institute of Anatomy, Faculty of Medicine, University of Ljubljana, 1000 Ljubljana, Slovenia

**Keywords:** Fascia, obesity, hyaluronan, fasciopathy, myofascial disease, pathophysiology, therapeutics

## Abstract

Myofascial disease is an important complication associated with obesity and one of the leading causes of physical disability globally. In the face of limited treatment options, the burden of myofascial disorders is predicted to increase along with the escalating prevalence of obesity. Several pathological processes in obesity contribute to modifications in fascial extracellular matrix mechanical and biological properties and functions. Changes in adipose tissue metabolism, chronic inflammatory phenotype, oxidative stress, and other mechanisms in obesity may alter the physiochemical and biomechanical properties of fascial hyaluronan. Understanding the pathophysiological importance of hyaluronan and other components of the fascial connective tissue matrix in obesity may shed light on the etiology of associated myofascial disorders and inform treatment strategies. Given its unique and favorable pharmacological properties, hyaluronan has found a broad range of clinical applications, notably in orthopedic conditions such as osteoarthritis and tendinopathies, which share important pathophysiological mechanisms implicated in myofascial diseases. However, while existing clinical studies uniformly affirm the therapeutic value of hyaluronan in myofascial disorders, more extensive studies in broader pharmacological and clinical contexts are needed to firmly validate its therapeutic adaptation.

## 1. Introduction

The Fascia Research Society, through its Fascia Nomenclature Committee, has proposed both anatomical and functional definitions of the fascia. Morphologically, a fascia was defined as “a sheath, a sheet, or any other dissectible aggregations of connective tissue that forms beneath the skin to attach, enclose, and separate muscles and other internal organs”. Functionally, the fascial system was defined as “the three-dimensional continuum of soft, collagen-containing, loose and dense fibrous connective tissues that permeate the body, providing an environment that enables all body systems to operate in an integrated manner” [1,2,3]. Based on histological properties and anatomical relationships, fascia may be classified into four types: superficial (subcutaneous) fascia, deep/muscular fasciae (aponeurotic and epimysial fasciae), visceral fasciae, and neural fasciae (meningeal layers and connective tissue sheath of peripheral nerves) [4,5]. Structurally the fascia tissue consists of various cell types (fibroblasts, myofibroblasts, fasciacytes, and telocytes), an extracellular matrix consisting of fibrous (types I and III collagen fibers, elastin, and fibrillin), and aqueous (water and complex mixture of glycosaminoglycans) components, and nerve elements (free nerve endings and mechanoreceptors) [4,6].

While the biomechanical properties of the clinically important fascia, such as the plantar fascia, have been well-studied [7,8,9,10], the microscopic anatomy and pathology of the fascia have received limited attention. Little is known about the elastic fiber composition, extracellular matrix characteristics, vascularity, innervation extent of the fascia tissue, and their role in disease and therapeutics [11]. Deepening our understanding of the microanatomical and biochemical basis of fascial disease in obesity may provide novel therapeutic insights for the medical and surgical treatment of obesity-related myofascial complications. A significant body of evidence has highlighted the critical role of fascial cells, extracellular matrix, and nerve elements in the pathogenesis of myofascial disease [4,6]. In this regard, a therapeutically relevant consideration is the role of hyaluronan (hyaluronic acid) in clinical fasciopathy. The present review aims to examine the current evidence on the pathological role and therapeutic potential of fascial hyaluronan in obesity-related myofascial disorders.

## 2. Hyaluronan Biochemistry, Cellular Synthesis, and Homeostasis

Despite being first described nearly 90 years ago, the role of hyaluronan in fascia physiology and pathology has only received focal attention in recent decades [12,13]. Hyaluronan is the dominant polysaccharide of the extracellular matrix of connective tissues with high cross-species structural homology, being structurally identical in bacteria and vertebrates [14]. It can be found in connective, epithelial, and neural tissues, where it provides mechanical stability and acts as a water reservoir, lubricant, and extracellular matrix homeostatic regulator [15]. Besides fascial hyaluronan, other body tissues such as the skin, tendon sheaths, pleura, pericardium, synovial fluid, the vitreous body, and the umbilical cord are also rich in hyaluronan. A 70-kg body has 15 g of hyaluronan, and about 50% of total body hyaluronan is located in the dermis [14,16].

Hyaluronan is a linear non-sulfated glycosaminoglycan composed of a single polysaccharide chain built by repeated disaccharide units of N-acetyl-D-glucosamine and D-glucuronic acid, respectively linked by β1–3 and β1–4 glycosidic bonds [12]. It is synthesized by three plasma membrane-bound hyaluronan synthases (HAS1, HAS2, and HAS3). As hyaluronan is synthesized in the plasma membrane rather than the Golgi, it lacks peptides in its fundamental structure, unlike other glycosaminoglycans [17]. Hyaluronan may be found in tissues in three forms: attached to plasma membranes, aggregated with other organic molecules, or as unbound polysaccharides [17]. Hyaluronan can interact with several extracellular matrix-binding proteins, such as aggrecans, but when not coupled to other molecules, it forms a viscous environment by self-associating and binding to water molecules [18]. In vivo, hyaluronan polymers range in size from 5000 to 20,000,000 Da and are divided into high and low molecular weight hyaluronan, each having different functional properties [19]. While high-molecular-weight hyaluronan contributes to tissue homeostasis by inhibiting cell proliferation, migration, angiogenesis, inflammation, and immunogenicity, hyaluronan oligomers have been shown to stimulate endothelial proliferation and migration, including tumor cell motility via their interaction with cluster determinant 44 (CD44) receptors, which is currently thought to be the major hyaluronan receptor on most cell types [20,21,22]. The membrane-bound CD44 regulates adhesion, motility, and intracellular signaling, while the receptor for hyaluronan-mediated motility (RHAMM) modulates intracellular signaling [23]. RHAMM is a centrosome- and microtubule-associated protein that is highly expressed before and during mitosis and hence prominent in neoplastic and other hyperproliferative tissues [24]. Besides the widely distributed CD44, hyaluronan fragments may also activate RHAMM, LYVE-1 (lymphatic vessel endothelial hyaluronan receptor), HARE (hyaluronan-receptor for endocytosis), ICAM-1 (intercellular adhesion molecule 1), layilin, Toll-like receptor 4, and other cell surface receptors which modulate gene expression via signaling pathways [23,25]. Accordingly, hyaluronan binding is critical for morphogenesis, matrix organization, wound repair/regeneration, inflammation, and metastasis [23].

While the predominant cell type in the fascia is the fibroblast which plays critical roles in mechanotransduction and synthesis of extracellular matrix precursors, a new class of previously undescribed cells termed “fasciacytes”, which are modified fibroblast-like cells located at the border of the different fascial layers, have been proposed as the site of fascial hyaluronan synthesis and secretion [4,26]. These cells are termed synoviocytes in the joints and hyalocytes in the eye, where they respectively secrete the hyaluronan of the synovial and vitreous fluids. Fasciacytes stain prominently with Alcian blue and are visualized as small clusters of rounded cells with circular nuclei, perinuclear cytoplasm, and small, less-elongated cellular processes [26]. They have been shown to express hyaluronan synthase 2 mRNA and are positive for the fibroblast marker vimentin, and negative for anti-CD68 indicating they are non-derivatives of the monocyte/macrophage lineage [26,27]. The deep muscular fascia expresses high levels of hyaluronan in the interface between the fascia and the epimysium [28]. This layer of hyaluronan-rich loose connective tissue between the deep fascia and the underlying skeletal muscle was also demonstrated by Stecco and colleagues using a combination of histological and sonographic analysis [29]. Hyaluronan has been shown to facilitate the gliding between different fascial sublayers and between fascia and muscle [28,30]. Given the evidence on its endomysial histolocalisation, it has also been suggested that hyaluronan not only lubricates but promotes muscle fiber motility [31].

Proteoglycans/glycosaminoglycans, elastin, fibronectin, laminin, and numerous other glycoproteins make up the thick dynamic extracellular matrix surrounding all fascia cells [4]. Fascia homeostasis is the outcome of dynamic interactions between cellular components and the extracellular matrix, and reciprocally, small extracellular matrix functional and structural changes contribute to complex cellular adaption mechanisms [32]. Additionally, the extracellular matrix functions as a molecular storage system, capturing and releasing physiologically active chemicals that govern cellular and tissue function, development, regeneration, and repair [33]. The mechanical properties of hyaluronan are determined by its molecular weight, tissue concentration, pH, covalent modifications, alterations in binding interactions with other molecules, and fluid dynamics [30,34]. The tissue half-life of hyaluronan ranges from a few hours to several days, and removal occurs by receptor-mediated endocytosis and lysosomal breakdown with subsequent elimination via lymph nodes, liver, and kidney [17,18]. Tissue hyaluronan equilibrium is maintained by both hyaluronan synthases and the cleavage enzymes, hyaluronidases (HYAL 1, 2, and 3), as well as non-enzymatic degradation via thermal or shear stress, acidic/alkaline hydrolysis, and reactive oxygen species [14,34]. Tissue volume, viscosity, and elasticity are all affected when hyaluronan mass or molecular weight decreases due to degradation or a decline in synthesis [18].

Healthy fascia requires a specific level of the lubricating layer of hyaluronan, which allows sliding between fascial sublayers and between fasciae and adjacent structures [35,36]. The hyaluronan composition of human fascial samples obtained from different anatomic regions was first quantified by Fede and colleagues in 2018 [36]. They demonstrated that hyaluronan concentration varies in accordance with the degree of fascial plane sliding and gliding functions in different anatomic regions. For example, the average hyaluronan concentration in the retinacula of the ankle (fascia associated with a mobile joint) was 90 μg/g of fascial tissue, in contrast to the fascia adherent to a muscle (epimysial fascia), with limited lubrication requirement, such as fascia overlying the trapezius and deltoid, which had an average hyaluronan content of 6 μg/g of fascial tissue [36].

## 3. Obesity and Myofascial Disease

The global prevalence of obesity has escalated to pandemic proportions over the past half-century [37,38,39], with recent data from the World Health Organization revealing that the condition affects 13% of the world’s population [40]. A recent estimate of the economic impact of obesity in eight countries reported that the condition costs between 0.8% and 2.4% of gross domestic product (GDP) and that the magnitude of economic impact was similarly substantial in both low-, middle-, and high-income countries, and projected to increase if current trends persist [41]. The rising global trend of obesity is associated with the increasing prevalence of cardiometabolic disorders such as type 2 diabetes mellitus and hypertension, as well as a broad spectrum of orthopedic morbidities [42,43]. A growing body of evidence suggests that the histological and biomolecular changes of the human fascia contribute to several myofascial and other connective tissue disorders associated with obesity and metabolic syndrome, including adhesive capsulitis, Dupuytren’s contracture, crystal-induced arthritis, plantar fasciitis, plantar fascia rupture, plantar fibromatosis, plantar xanthoma, and enthesopathy [12,29,43,44].

Myofascial pain syndromes are musculoskeletal pain disorders with a commonly associated neuropathic component and represent a leading cause of physical disability globally. They are thought to originate from myofascial trigger points, which are palpable hyperirritable painful spots involving a select number of muscle fibers, and may be acute or chronic, primary, or secondary to another comorbidity [45]. It was long assumed that the syndrome exclusively involved muscles, but current research suggests that the fascia plays a critical role, although the exact mechanisms and therapeutic significance of fascial pathophysiological roles remain an open subject for further investigation [46]. Pathological degenerative changes in the fascia, or fasciitis, are among the leading causes of functionally limiting musculoskeletal pain syndromes. For example, plantar fasciitis affects one in ten people in their lifetime and accounts for 1% of all orthopedic consultations [47]. A high prevalence of myofascial pain (high proportion of latent and active myofascial trigger points) is similarly reported in patients presenting with chronic back pain, non-specific neck pain, and chronic non-traumatic shoulder pain [48,49,50,51]. The prevalence of myofascial pain ranges from 21–93% in general orthopedic practice and specialist pain clinic patients, respectively, and up to 85% of people in the general population will experience myofascial pain in their lifetime [52]. In the United States alone, the national economic burden of plantar fasciitis was estimated at 284 million US dollars, with medication costs accounting for about 80% of total costs [53]. Several risk factors have been identified, including obesity, traumatic musculoskeletal injuries, spine disease, cumulative and repeated strain, postural dysfunction, and physical deconditioning [45].

Obesity is a key epidemiological risk factor for myofascial disease [54,55,56,57,58,59,60]. A systematic review of 51 studies found that the only significant predictor related to plantar fasciitis was a body mass index (BMI) >27 kg/m^2^ [61]. Even in an active population such as recreational and competitive runners, of eleven analyzed risk factors, increased BMI and body mass were found to be primary risk factors for fasciopathy [55]. In recent-onset type 2 diabetic subjects without complications, plantar fascia thickness was increased compared to the controls and significantly associated with adiposity and BMI values, suggesting important clinical implications in obese diabetic patients [62]. Plantar fascia thickness has also been shown to be a reliable alternative index of tissue glycation and a significant predictor of microvasculopathy, an essential denominator in several obesity-related complications [63,64,65,66,67]. Cytokines and other inflammatory molecules that have been demonstrated in the environment of myofascial trigger points are also typically overexpressed in the skeletal muscle of obese patients [68,69]. High-fat diet-induced obese mice were shown to express elevated spontaneous neurotransmission, which facilitates the development of myofascial trigger points [60].

## 4. Pathophysiological Importance and Associated Alterations of Hyaluronan in Obesity

Hyaluronan dysregulation has been implicated in the pathophysiology of several clinical conditions, including cancer, diabetes, autoimmune disease, and vascular disease [70,71,72,73,74,75,76]. Similarly, hyaluronan-mediated signaling is disrupted in various tissues in obesity. In metabolic comorbidities associated with obesity, such as nonalcoholic hepatic steatosis and insulin resistance, elevated circulatory levels of hyaluronan have been demonstrated and suggested to have diagnostic value [77,78,79,80].

Hyaluronan binds to cell-surface proteins, including receptors, to exert a broad range of biological effects on adipose tissue. Increasing evidence points to the involvement of hyaluronan and its receptors in obesity-related adipocyte hyperplasia and hypertrophy and adipose tissue metabolism [81]. An enhanced expression of hyaluronan synthase-1 was demonstrated in adipose tissue from obese patients [82]. It has been suggested that adipocyte hypertrophy contributes to adipose tissue inflammation [83]. Adipose tissue hypertrophy during obesity-related weight gain severely destabilizes extracellular matrix homeostasis by modifying the local oxygen supply, which in turn triggers cellular stress events and inflammation [82]. A potential role of hyaluronan in adipogenesis in vivo has been demonstrated in mouse models of high-fat diet-induced obesity [79,84,85,86,87]. Hyaluronan levels were noted to be increased in these mice, mediating insulin resistance via CD44-dependent mechanisms. Treatment with exogenous hyaluronidase was found to dose-dependently decrease fat mass and adipocyte size and inhibit abdominal, muscular, and hepatic lipid accumulation, consequently increasing insulin sensitivity [84,85,86].

The accumulation and turnover of hyaluronan polymers in many cell types have been linked to inflammation. The role of hyaluronan in regulating inflammatory responses, including the expression of inflammatory genes, the recruitment of inflammatory cells, and the production of inflammatory cytokines, is now firmly established [88]. Hyaluronan modulates the cellular proliferative phase of tissue repair following inflammatory damage by facilitating fibroblast detachment from the extracellular matrix, mitosis, and cell migration via CD44 and RHAMM interactions [89,90,91]. Human and animal inflammatory disorders such as sarcoidosis, idiopathic pulmonary fibrosis, farmer’s lung, graft rejection, experimental myocarditis, myocardial infarction, and inflammatory bowel disease are associated with increased hyaluronan levels in tissues [92]. As previously noted, high-molecular-weight hyaluronan is anti-inflammatory and immunosuppressive, while low-molecular-weight hyaluronan is pro-inflammatory [21,22].

It was shown that while total plasma hyaluronan molecules remain unaltered, the circulating level of low-molecular-weight hyaluronan fragments is elevated in obesity and may play a key role via Toll-like receptor (TLR)-mediated activation of innate immune cells in activating low-grade inflammatory phenotypes and other metabolic complications [82,93]. The expression of hyaluronan receptors in leukocytes is altered in obesity, with consequent alterations in the inflammatory response of leucocytes to low-molecular-weight hyaluronan. It was shown that low-molecular-weight hyaluronan induces nuclear factor kappa B (NF-κB)-dependent activation in peripheral blood monocytes and THP-1 monocytes, leading to an increase in pro-inflammatory markers [82]. Hyaluronan increases tumor necrosis factor-α (TNF-α), insulin-like growth factor-1 (IGF-1) mRNA transcript expression and protein synthesis, interleukin 1 beta (IL-1β), and interleukin 8 (IL-8) via a CD44-mediated mechanism and modulates cytokine-activated lymphocyte adhesion to the endothelium [94,95]. TNF-α may also trigger the release of the hyaluronan-binding protein, TSG-6 (TNFα-stimulated gene-6 protein), which propagates the inflammatory response [96], while IL-1β up-regulates hyaluronan synthase-1 gene expression in adipose tissue [82].

The inflammatory milieu in obesity underlies the pathophysiology of several associated complications, including the development of fasciopathies and tendinopathies [97,98,99,100,101]. The array of proinflammatory mediators, such as cytokines, adipokines (e.g., leptins), lipocalin-1, serum amyloid A-3, and adiponectin released during the development of obesity-related chronic inflammatory phenotypes promote insulin resistance by altering the extracellular matrix, the capillary network architecture, and the glucose uptake mechanisms [102]. The resulting hyperglycemia causes changes in the stiffness, gliding, and distribution of force transmission in the fasciae due to collagen thickening, elastic fiber fragmentation, and changes in glycosaminoglycans, particularly hyaluronan, with cascading ramifications at the cellular and molecular levels, including alterations in cellular proliferation, differentiation, growth, and migration [4]. In addition, inflammation increases reactive oxygen species, which degrade collagen, laminin, and hyaluronan, and hyaluronan fragments generated by this process sustain an inflammatory cycle (recruitment of leukocytes and release of various inflammatory mediators such as reactive oxygen species, cytokines, chemokines, and destructive enzymes) [14,88]. Conversely, high molecular weight hyaluronan works as an effective barrier to the inflammatory process and protects against oxidative damage by free radicals (superoxide anions, hydroxyl radicals, and hypochlorite) [103].

## 5. The Etiological Significance of Changes in Hyaluronan Properties in Myofascial Disease

**Figure 1:**
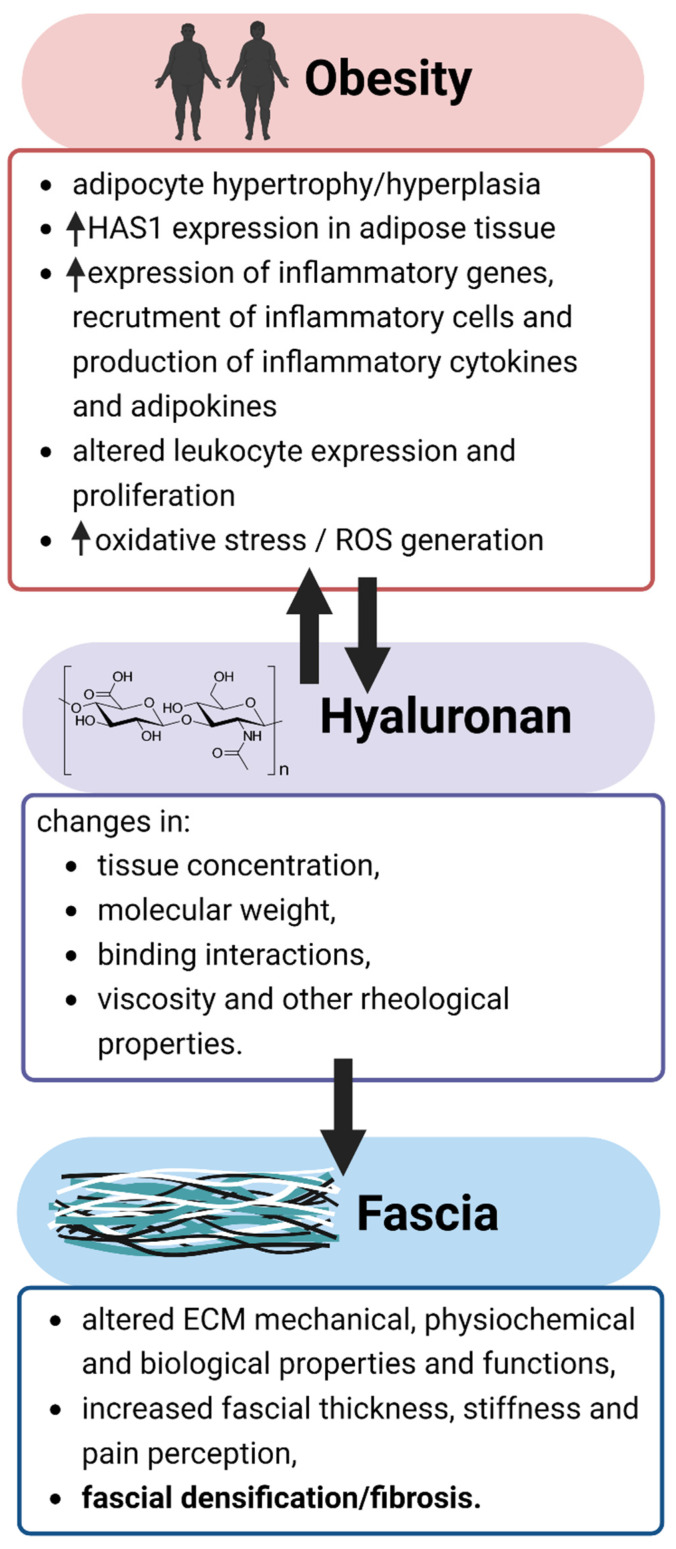
Relationships of pathophysiological mechanisms in obesity with changes in hyaluronan properties and the development of myofascial disease. ECM: extracellular matrix; HAS1: hyaluronan synthase 1; ROS: reactive oxygen species.

The fibrous and glycosaminoglycan components of the fascial extracellular matrix can be affected by various physical, mechanical, hormonal, and pharmacological factors [4]. Alterations in the physiological levels of hyaluronan have been demonstrated to be etiologically important in myofascial pain syndromes [29,36]. Changes in the physical and chemical properties of hyaluronan are associated with modifications in extracellular matrix viscoelasticity, mechanical plasticity, and nonlinear elasticity [104,105], all of which may contribute to myofascial disease (Figure 1). Although the evidence is conflicting, it has been suggested that a strong association exists between body temperature and obesity markers [106]. With increasing temperature, both stiffening and weakening of hyaluronan-based hydrogels were observed [107,108]. Given that pH is directly related to viscosity [109], the biomechanical properties of hyaluronan may be altered by tissue acidity from increased lactic acid accumulation seen in obesity [110]. It was shown that hyaluronan degradation occurs at pH < 4 and pH > 11 [111]. Alterations in hyaluronan function may result from the effects of van der Waals and hydrophobic forces on its concentration, polyelectrolyte properties, and aggregation characteristics [25,108]. Hyaluronan takes on non-Newtonian characteristics and becomes more viscous at higher concentrations [112]. Myofascial disorders may originate from altered hydrodynamic characteristics and atypical viscoelastic properties of fascia [46]. Obesity is associated with diminished physical mobility [113,114], which has been shown to raise the concentration of hyaluronan without adequate hyaluronan recycling, increase hyaluronan viscosity, and limit the lubrication and gliding of the layers of connective tissue and muscle, with a consequent increase in overall fascial thickness, stiffness, and pain perception [30]. Any loading condition reduces hyaluronan viscosity; however, resting conditions allow hyaluronan to recover to a more viscous state.

The distribution of lines of force inside the fascia alters when hyaluronan changes from lubricating to adhesive function, a process referred to as densification of fascia [115,116]. As the connective tissue and its extracellular matrix thicken and become denser, the capacity of fascial tissue to slide is reduced or eliminated [116,117]. Chronic densification modifies the gliding of the fibrous layers, influencing collagen fiber deposition locally and remotely [116]. While fascial densification and fibrosis describe alterations in the fascia resulting in myofascial pain syndromes, it is important to differentiate both processes as they have distinct pathological and therapeutic implications. Densification indicates a potentially easily reversible modification in the loose connective tissue due to hyaluronan super-aggregation with a decreased water-binding capacity resulting in altered mechanical characteristics of the fascia but not its overall structure, whereas fibrosis refers to a difficult-to-reverse modification of the general tissue structure and mechanical properties from the excessive deposition of fibrous connective tissue as part of a reparative or reactive response [25,116]. The loose connective tissue found within the deep fascia can be altered by diet, exercise, and overuse syndromes, resulting in fascial densification, whereas trauma, surgery, and diabetes can modify the fibrous layers of the deep fasciae, resulting in fibrosis of the fascia [116]. Chronic, nonspecific neck pain may be reflective of fascial densification, whereas Dupuytren’s disease and eosinophil fasciitis are typical fibrotic disorders, and therapeutic approaches are distinct in both pathologies [46,118]. Studies have demonstrated that mechanosensitive signaling underlies obesity-induced connective tissue fibrosis [119]. Fascial tissues contain various types of mechanoreceptors, in addition to the vast network of free nerve endings that play important roles in pain perception and regulation [120,121]. Myofibroblasts in the fascia, which are specialized fibroblasts with contractile properties that regulate the tissue basal tone, are also etiologically important in some pathological fibrotic contractures such as Dupuytren disease that affects the palmar and digital fascia of the hand [4].

## 6. Therapeutic Considerations of Hyaluronan in Myofascial Disease

The complicated peripheral and central pathophysiological mechanisms in myofascial pain syndromes present unique challenges for effective treatment. Current pharmacological therapies include nonsteroidal anti-inflammatory drugs (NSAIDs), opioid analgesics (e.g., tramadol), muscle relaxants (e.g., tizanidine, cyclobenzaprine), anticonvulsants (e.g., gabapentin and pregabalin), antidepressants (e.g., tricyclic antidepressants such as amitriptyline and serotonin-norepinephrine reuptake inhibitors such as duloxetine), benzodiazepines, tropisetron (5-HT_3_ receptor antagonist and alpha-7-nicotinic receptor agonist), sumatriptan (peripheral 5-HT receptor agonist), lidocaine transdermal patch, intramuscular ketamine, steroid injections, and botulinum type A toxin (BoNT-A) injections. Several non-pharmacological treatment modalities have also been proposed, including manual therapy, dry needling, ultrasound therapy, ischemic compression, phonophoresis, pressure release, transcutaneous electric nerve stimulation (TENS), electrical twitch obtaining intramuscular stimulation (ETOIMS), magnetic stimulation, and laser therapy [52,122,123,124,125,126]. Unfortunately, the current pharmacological and non-pharmacological treatment modalities are not backed by high-quality evidence regarding efficacy and safety, and the search for evidence-based effective treatment options continues.

Unraveling novel therapeutic approaches to alleviate chronic pain syndromes may be facilitated by an enhanced understanding of the pathophysiological importance of hyaluronan and other components of the connective tissue matrix of fascia, as well as the mechanical forces that are both permitted and restricted by fascial planes [116]. Hyaluronan’s ubiquitous availability, complete resorbability, biocompatibility, hydrophilicity, unique viscoelasticity, and minimal immunogenicity and adverse effects account for its wide biomedical and clinical applications in different fields of medicine (e.g., viscosupplementation for osteoarthritis treatment, vitreous substitution/replacement in ophthalmic surgery, as dermatological fillers, as scaffolds in nerve-, vessel-, and adipose tissue- engineering, as drug conjugation and delivery agent, and as an immunomodulatory agent in cancer therapeutics) [14,18,127,128,129]. Cosmetic injection of hyaluronan as a dermal filler (an FDA-approved clinical use) was ranked as the second and third most common non-surgical procedure for women and males, respectively [130,131]. Intra-synovial injection of crosslinked and non-crosslinked hyaluronan as viscosupplements is also a favored and FDA-approved treatment for osteoarthritic pain [132,133,134].

Several preclinical and clinical studies have reported on the therapeutic applications of hyaluronan in managing fasciopathies, tendinopathies, and osteoarthritis, all of which share important pathophysiological mechanisms [18,135,136,137,138,139,140,141,142,143,144,145,146]. In an animal model of osteoarthritis, administration of hyaluronan to isolated medial articular nerves dramatically lowered both ongoing and movement-evoked nerve activities, indicating a therapeutically important antinociceptive activity in inflamed joints through an elastoviscous, rheological effect on nociceptive afferent fibers [147]. In vitro experiments have demonstrated therapeutically important effects of hyaluronan on the extracellular matrix in osteoarthritis, including increased synthesis of chondroitin sulfate and proteoglycans, suppressed proteoglycan release from chondrocyte, and cartilage cell-matrix, and inhibited proteoglycan breakdown from cartilage. Several effects of hyaluronan on inflammatory mediators and immune cells have been described, notably decreased levels of IL-1-induced prostaglandin E_2_, TNF-α, plasminogen activator activity, increased tissue inhibitor of metalloproteinases-1, enhanced antioxidant effects, reduced lymphocyte stimulation, motility, and proliferation, suppressed neutrophil aggregation and adhesion, inhibited macrophage and neutrophil phagocytosis, and enhanced polymorphonuclear leukocyte phagocytosis, adherence, and migration [148]. These effects of hyaluronan on the extracellular matrix, inflammatory mediators, and immune cells are therapeutically important in fascial disease and support the adaptability of hyaluronan for the treatment of myofascial disorders. As in osteoarthritis, functionally limited patients with myofascial disease who have not responded adequately to conventional pharmacological and nonpharmacological treatment options, those who have gastrointestinal or renal intolerance to NSAIDs and other therapies, and those who wish to postpone or are ineligible for surgery are good candidates for hyaluronan treatment [132]. On the other hand, it has been suggested that obesity may be an independent risk factor for viscosupplementation failure in patients with osteoarthritis [149], although further investigation demonstrated that benefits were similar in normal-weight and obese patients with mild or moderate knee osteoarthritis who responded to treatment [150].

Indeed, a potential therapeutic role of hyaluronan injections in treating fasciopathies has been demonstrated [151]. A recent randomized controlled trial found that the administration of five injections of high-molecular-weight hyaluronan is a safe and effective treatment option for patients with persistent pain for more than 12 weeks from plantar fasciopathy [152]. In patients with various enthesopathies (lateral epicondylitis, patellar tendinopathy, insertional Achilles tendinopathy, and plantar fasciitis), a single injection of up to 2.5 mL hyaluronan uniformly reduced pain as assessed by the visual analog scale (VAS) for pain and local pain symptoms 1 week after injection [145]. Tendinopathies and fasciopathies share similar pathophysiological mechanisms, mindful that tendons are technically part of the fascial system. Recall that the broader functional definition of the fascial system incorporates elements such as adipose tissue, adventitiae and neurovascular sheaths, aponeuroses, deep and superficial fasciae, epineurium, joint capsules, ligaments, membranes, meninges, myofascial expansions, periostea, retinacula, septa, tendons, and visceral fasciae [1].

A few studies have compared hyaluronan and other conventional therapies, as well as different pharmacological preparations and modifications of hyaluronan. Raeissadat et al. found that while corticosteroid injection appeared to have a faster trend of improvement in the short term, hyaluronan injection was comparably effective in reducing the symptoms of plantar fasciitis [144]. Additionally, hyaluronan may also be considered a physiologically more favorable option than corticosteroids which are notorious for a broad spectrum of short- and long-term adverse effects. At three months post-treatment, pain ratings indicated that two peritendinous hyaluronan injections were more effective than conventional extracorporeal shock wave therapy in treating patients with Achilles’ midportion tendinopathy for ≥6 weeks [153]. In patients with lateral elbow, Achilles, and patellar tendinopathy, ultrasound-guided peritendinous injections of lower-molecular-weight hyaluronan (500–730 kDa) were safe, well-tolerated, and associated with significant pain improvement and reduction in ultrasound-assessed tendon thickness and neovascularization [141]. In a recent study comparing ultrasound-guided injections with low molecular weight (500–730 KDa) and high molecular weight (>2000 KDa) hyaluronan, Mohebbi et al. found that while both hyaluronan types had similar efficacy in the treatment of rotator cuff tendinopathy, patients tolerated low-molecular-weight injections better [154]. In patients with knee osteoarthritis, Bahrami et al. showed that a single high-molecular-weight hyaluronan injection is as effective as multiple injections of low-molecular-weight hyaluronan at 2- and 6-months follow-up [155]. In contrast to the native form of hyaluronan (>1000 kDa), lower-molecular-weight forms of the molecule (<500 kDa, especially preparations in 100–250 kDa) are pathophysiologically important as proinflammatory mediators [156,157]. Thus, further studies will be needed to clarify the impact of molecular weight on the therapeutic effects of hyaluronan. Furthermore, mindful that most of the current pharmacologic adaptations of hyaluronan in myofascial disease primarily aim to address the pathological consequences of the changes in its tissue concentration or molecular weight, it would be useful to explore the therapeutic endogenous or exogenous modifications of hyaluronan to target other pathophysiological mechanisms such as covalent modifications, binding interactions, rheological alterations, pH changes, and alterations in adipose tissue metabolism and inflammatory phenotype. Focal studies in obese patients with myofascial disease are also warranted to clarify the impact of obesity on the therapeutic potential of hyaluronan in myofascial disease.

Some investigators have explored molecular alterations to exogenously administered hyaluronan to change the extracellular matrix’s mechanics. Low-load elastic mechanical features, such as lower toe modulus and a tendency toward lower toe stiffness and a larger transition strain, are seen in the fascial extracellular matrix treated with tyramine-substituted high-molecular-weight hyaluronan [158]. Given its relatively short half-life in biological fluids, a number of modifications of hyaluronan molecules to extend the duration of its biological actions have been explored. The most common of these is crosslinking to form a hydrogel; however, this could lead to higher viscosity and toxicity [159]. On the other hand, stimulating endogenous hyaluronan production has been explored as another therapeutic strategy. The endocannabinoid receptors 1 and 2 (CB1 and CB2) are expressed in various deep fasciae, and it is known that the endocannabinoid system is crucially involved in the modulation of pain, inflammation, and fibrosis [160]. For example, a synthetic cannabinoid induced a rapid production of hyaluronan and hyaluronan-rich vesicles in an in vitro culture of fascial fibroblasts, confirming a peripheral effect of the endocannabinoid system on fascial cell regulation and remodeling of the formation of the extracellular matrix [143].

A potential therapeutic role of exogenous hyaluronidases has also been suggested. Trigger point injection of hyaluronidase was reported to substantially decrease clinically assessed pain in patients with myofascial pain disorders [161]. Similarly, in patients with spastic disorders partly related to hyaluronan accumulation in muscles, hyaluronidase injections improved both passive and active mobility [162]. It is thought that the beneficial effects may be related to modifications in hyaluronan viscosity. In addition, recombinant hyaluronidase PH20 (PEGPH20) was found to significantly reduce adipose tissue mass and adipocyte size and improve insulin sensitivity in a mouse model of diet-induced obesity [79]. These effects may ameliorate the pathological ramifications of adipose tissue inflammation in myofascial disease. Finally, an understanding of the rheological properties of hyaluronan provides a physiochemical explanation of the therapeutic effects of massage, manipulation, laser therapy, and other physical therapy procedures in myofascial disease, namely the disaggregation of the pathologic chain–chain interactions of hyaluronan molecule under controlled physical conditions such as high temperature [46].

## 7. Conclusions

Obesity is a primary risk factor for myofascial disease, which is a leading cause of physical disability globally. With the escalating prevalence of obesity, it is expected that the burden of myofascial disease will worsen. Unfortunately, current treatment options are limited in terms of safety and efficacy, and thus new therapeutic strategies are needed. Clarifying the etiological significance of various components of the connective tissue matrix of the fascia in obesity may aid in developing novel therapeutic methods for obesity-related myofascial disorders. In particular, understanding the pathophysiological importance of fascial hyaluronan may provide valuable therapeutic insights. Hyaluronan is the dominant polysaccharide of the extracellular matrix of connective tissues, which provides mechanical stability and acts as a water reservoir and lubricant, allowing sliding between fascial sublayers and between fasciae and adjacent structures. It also acts as an extracellular matrix homeostatic regulator via various cellular mechanisms and interactions.

Alterations in the physical and chemical properties of hyaluronan are associated with modifications in extracellular matrix viscoelasticity and other mechanical properties and biological functions and have been demonstrated as critical factors in the development of myofascial disease in obesity. Understanding this pathophysiological connection paves the way for the potential therapeutic exploitation of hyaluronan. Given its ubiquitous availability and unique pharmacological properties, hyaluronan has found a broad range of clinical applications, including in orthopedic disorders such as osteoarthritis, tendinopathies, and fasciopathies. While the relatively few results of therapeutic adaptation in myofascial disease are promising and supported by the current understanding of the pathophysiological importance of hyaluronan in fasciopathy, larger and more rigorous clinical trials utilizing a broader spectrum of myofascial disorders or different modifications of hyaluronan molecule are warranted to validate its therapeutic potential.

## Data Availability

Not applicable.

## References

[1] Adstrum S., Hedley G., Schleip R., Stecco C., Yucesoy C.A. (2017). Defining the Fascial System. J. Bodyw. Mov. Ther..

[2] Bordoni B., Escher A.R., Tobbi F., Pianese L., Ciardo A., Yamahata J., Hernandez S., Sanchez O. (2022). Fascial Nomenclature: Update 2022. Cureus.

[3] Schleip R., Hedley G., Yucesoy C.A. (2019). Fascial Nomenclature: Update on Related Consensus Process. Clin. Anat..

[4] Fede C., Pirri C., Fan C., Petrelli L., Guidolin D., de Caro R., Stecco C. (2021). A Closer Look at the Cellular and Molecular Components of the Deep/Muscular Fasciae. Int. J. Mol. Sci..

[5] Bordoni B., Varacallo M. (2018). Anatomy, Fascia. StatPearls.

[6] Pirri C., Fede C., Pirri N., Petrelli L., Fan C., de Caro R., Stecco C. (2021). Diabetic Foot: The Role of Fasciae, a Narrative Review. Biology.

[7] Wang M., Li S., Teo E.C., Fekete G., Gu Y. (2021). The Influence of Heel Height on Strain Variation of Plantar Fascia During High Heel Shoes Walking-Combined Musculoskeletal Modeling and Finite Element Analysis. Front. Bioeng. Biotechnol..

[8] Schleip R., Klingler W. (2019). Active Contractile Properties of Fascia. Clin. Anat..

[9] White A., Abbott H., Masi A.T., Henderson J., Nair K. (2018). Biomechanical Properties of Low Back Myofascial Tissue in Younger Adult Ankylosing Spondylitis Patients and Matched Healthy Control Subjects. Clin. Biomech..

[10] Schleip R., Gabbiani G., Wilke J., Naylor I., Hinz B., Zorn A., Jäger H., Breul R., Schreiner S., Klingler W. (2019). Fascia Is Able to Actively Contract and May Thereby Influence Musculoskeletal Dynamics: A Histochemical and Mechanographic Investigation. Front. Physiol..

[11] Stecco C., Corradin M., Macchi V., Morra A., Porzionato A., Biz C., de Caro R. (2013). Plantar Fascia Anatomy and Its Relationship with Achilles Tendon and Paratenon. J. Anat..

[12] Pratt R.L. (2021). Hyaluronan and the Fascial Frontier. Int. J. Mol. Sci..

[13] Meyer K., Palmer J.W. (1934). The Polysaccharide of the Vitreous Humor. J. Biol. Chem..

[14] Salwowska N.M., Bebenek K.A., Żądło D.A., Wcisło-Dziadecka D.L. (2016). Physiochemical Properties and Application of Hyaluronic Acid: A Systematic Review. J. Cosmet. Dermatol..

[15] Balazs E.A., Laurent T.C., Jeanloz R.W. (1986). Nomenclature of Hyaluronic Acid. Biochem. J..

[16] Keen M.A. (2017). Hyaluronic Acid in Dermatology. Skinmed.

[17] Fraser J.R.E., Laurent T.C., Laurent U.B.G. (1997). Hyaluronan: Its Nature, Distribution, Functions and Turnover. J. Intern. Med..

[18] Fakhari A., Berkland C. (2013). Applications and Emerging Trends of Hyaluronic Acid in Tissue Engineering, as a Dermal Filler and in Osteoarthritis Treatment. Acta Biomater..

[19] Ruckmani K., Shaikh S.Z., Khalil P., Muneera M.S., Thusleem O.A. (2013). Determination of Sodium Hyaluronate in Pharmaceutical Formulations by HPLC–UV. J. Pharm. Anal..

[20] Jacobetz M.A., Chan D.S., Neesse A., Bapiro T.E., Cook N., Frese K.K., Feig C., Nakagawa T., Caldwell M.E., Zecchini H.I. (2013). Hyaluronan Impairs Vascular Function and Drug Delivery in a Mouse Model of Pancreatic Cancer. Gut.

[21] Lee B.M., Park S.J., Noh I., Kim C.H. (2021). The Effects of the Molecular Weights of Hyaluronic Acid on the Immune Responses. Biomater. Res..

[22] Tavianatou A.G., Caon I., Franchi M., Piperigkou Z., Galesso D., Karamanos N.K. (2019). Hyaluronan: Molecular Size-Dependent Signaling and Biological Functions in Inflammation and Cancer. FEBS J..

[23] Entwistle J., Hall C.L., Turley E.A. (1996). HA Receptors: Regulators of Signalling to the Cytoskeleton. J. Cell Biochem..

[24] Jiang X., Tang L., Yuan Y., Wang J., Zhang D., Qian K., Cho W.C., Duan L. (2022). NcRNA-Mediated High Expression of HMMR as a Prognostic Biomarker Correlated with Cell Proliferation and Cell Migration in Lung Adenocarcinoma. Front. Oncol..

[25] Stecco A., Cowman M., Pirri N., Raghavan P., Pirri C. (2022). Densification: Hyaluronan Aggregation in Different Human Organs. Bioengineering.

[26] Stecco C., Fede C., Macchi V., Porzionato A., Petrelli L., Biz C., Stern R., de Caro R. (2018). The Fasciacytes: A New Cell Devoted to Fascial Gliding Regulation. Clin. Anat..

[27] Bartok B., Firestein G.S. (2010). Fibroblast-like Synoviocytes: Key Effector Cells in Rheumatoid Arthritis. Immunol. Rev..

[28] McCombe D., Brown T., Slavin J., Morrison W.A. (2001). The Histochemical Structure of the Deep Fascia and Its Structural Response to Surgery. J. Hand Surg. Br..

[29] Stecco C., Stern R., Porzionato A., MacChi V., Masiero S., Stecco A., de Caro R. (2011). Hyaluronan within Fascia in the Etiology of Myofascial Pain. Surg. Radiol. Anat..

[30] Cowman M.K., Schmidt T.A., Raghavan P., Stecco A. (2015). Viscoelastic Properties of Hyaluronan in Physiological Conditions. F1000Research.

[31] Piehl-Aulin K., Laurent C., Engstrom-Laurent A., Hellstrom S., Henriksson J. (1991). Hyaluronan in Human Skeletal Muscle of Lower Extremity: Concentration, Distribution, and Effect of Exercise. J. Appl. Physiol..

[32] Chen B., Ji B., Gao H. (2015). Modeling Active Mechanosensing in Cell-Matrix Interactions. Annu. Rev. Biophys..

[33] Zügel M., Maganaris C.N., Wilke J., Jurkat-Rott K., Klingler W., Wearing S.C., Findley T., Barbe M.F., Steinacker J.M., Vleeming A. (2018). Fascial Tissue Research in Sports Medicine: From Molecules to Tissue Adaptation, Injury and Diagnostics: Consensus Statement. Br. J. Sports Med..

[34] Wolf K.J., Kumar S. (2019). Hyaluronic Acid: Incorporating the Bio into the Material. ACS Biomater. Sci. Eng..

[35] Csoka A.B., Stern R. (2013). Hypotheses on the Evolution of Hyaluronan: A Highly Ironic Acid. Glycobiology.

[36] Fede C., Angelini A., Stern R., Macchi V., Porzionato A., Ruggieri P., de Caro R., Stecco C. (2018). Quantification of Hyaluronan in Human Fasciae: Variations with Function and Anatomical Site. J. Anat..

[37] Bentham J., di Cesare M., Bilano V., Bixby H., Zhou B., Stevens G.A., Riley L.M., Taddei C., Hajifathalian K., Lu Y. (2017). Worldwide Trends in Body-Mass Index, Underweight, Overweight, and Obesity from 1975 to 2016: A Pooled Analysis of 2416 Population-Based Measurement Studies in 128·9 Million Children, Adolescents, and Adults. Lancet.

[38] Reilly J.J., El-Hamdouchi A., Diouf A., Monyeki A., Somda S.A. (2018). Determining the Worldwide Prevalence of Obesity. Lancet.

[39] Yanovski J.A. (2018). Obesity: Trends in Underweight and Obesity—Scale of the Problem. Nat. Rev. Endocrinol..

[40] World Health Organization Obesity and Overweight. https://www.who.int/news-room/fact-sheets/detail/obesity-and-overweight.

[41] Okunogbe A., Nugent R., Spencer G., Ralston J., Wilding J. (2021). Economic Impacts of Overweight and Obesity: Current and Future Estimates for Eight Countries. BMJ Glob. Health.

[42] Afshin A., Forouzanfar M.H., Reitsma M.B., Sur P., Estep K., Lee A., Marczak L., Mokdad A.H., Moradi-Lakeh M., GBD 2015 Obesity Collaborators (2017). Health Effects of Overweight and Obesity in 195 Countries over 25 Years. N. Engl. J. Med..

[43] Paulis W.D., Silva S., Koes B.W., van Middelkoop M. (2014). Overweight and Obesity Are Associated with Musculoskeletal Complaints as Early as Childhood: A Systematic Review. Obes. Rev..

[44] Jannini S.N., Doŕia-Filho U., Damiani D., Silva C.A.A. (2011). Musculoskeletal Pain in Obese Adolescents. J. Pediatr..

[45] Weller J.L., Comeau D., Otis J.A.D. (2018). Myofascial Pain. Semin. Neurol..

[46] Stecco A., Gesi M., Stecco C., Stern R. (2013). Fascial Components of the Myofascial Pain Syndrome. Curr. Pain Headache Rep..

[47] Riddle D.L., Schappert S.M. (2004). Volume of Ambulatory Care Visits and Patterns of Care for Patients Diagnosed with Plantar Fasciitis: A National Study of Medical Doctors. Foot Ankle Int..

[48] Ezzati K., Ravarian B., Saberi A., Salari A., Reyhanian Z., Khakpour M., Chabok S.Y. (2021). Prevalence of Cervical Myofascial Pain Syndrome and Its Correlation with the Severity of Pain and Disability in Patients with Chronic Non-Specific Neck Pain. Arch. Bone Jt. Surg..

[49] Cerezo-Téllez E., Torres-Lacomba M., Mayoral-del Moral O., Sánchez-Sánchez B., Dommerholt J., Gutiérrez-Ortega C. (2016). Prevalence of Myofascial Pain Syndrome in Chronic Non-Specific Neck Pain: A Population-Based Cross-Sectional Descriptive Study. Pain Med..

[50] Bron C., Dommerholt J., Stegenga B., Wensing M., Oostendorp R.A. (2011). High Prevalence of Shoulder Girdle Muscles with Myofascial Trigger Points in Patients with Shoulder Pain. BMC Musculoskelet. Disord..

[51] Chen C.K., Nizar A.J. (2011). Myofascial Pain Syndrome in Chronic Back Pain Patients. Korean J. Pain.

[52] Desai M.J., Saini V., Saini S. (2013). Myofascial Pain Syndrome: A Treatment Review. Pain Ther..

[53] Tong K.B., Furia J. (2010). Economic Burden of Plantar Fasciitis Treatment in the United States. Am. J. Orthop..

[54] Rogers J.A., Jones G., Cook J., Squibb K., Wills K., Lahham A., Winzenberg T. (2021). Chronic Plantar Heel Pain Modifies Associations of Ankle Plantarflexor Strength and Body Mass Index with Calcaneal Bone Density and Microarchitecture. PLoS ONE.

[55] Hamstra-Wright K.L., Huxel Bliven K.C., Bay R.C., Aydemir B. (2021). Risk Factors for Plantar Fasciitis in Physically Active Individuals: A Systematic Review and Meta-Analysis. Sports Health.

[56] Choudhary R., Kunal K. (2021). Modifiable Risk Factors of Plantar Fasciitis in Non-Athletic Patients and Proposal of a New Objective Assessment System—RKISP. Rev. Bras. Ortop..

[57] Riddle D.L., Pulisic M., Pidcoe P., Johnson R.E. (2003). Risk Factors for Plantar Fasciitis: A Matched Case-Control Study. J. Bone Jt. Surg. Ser. A.

[58] Irving D.B., Cook J.L., Young M.A., Menz H.B. (2007). Obesity and Pronated Foot Type May Increase the Risk of Chronic Plantar Heel Pain: A Matched Case-Control Study. BMC Musculoskelet. Disord..

[59] Mickle K.J., Steele J.R. (2015). Obese Older Adults Suffer Foot Pain and Foot-Related Functional Limitation. Gait Posture.

[60] Gimenez-Donoso C., Bosque M., Vila A., Vilalta G., Santafe M.M. (2020). Effects of a Fat-Rich Diet on the Spontaneous Release of Acetylcholine in the Neuromuscular Junction of Mice. Nutrients.

[61] van Leeuwen K.D.B., Rogers J., Winzenberg T., van Middelkoop M. (2016). Higher Body Mass Index Is Associated with Plantar Fasciopathy/’plantar Fasciitis’: Systematic Review and Meta-Analysis of Various Clinical and Imaging Risk Factors. Br. J. Sports Med..

[62] Abate M., Schiavone C., di Carlo L., Salini V. (2012). Achilles Tendon and Plantar Fascia in Recently Diagnosed Type II Diabetes: Role of Body Mass Index. Clin. Rheumatol..

[63] Craig M.E., Cusumano J., Duffin A.C., Hing S., Gallego P.H., Donaghue K.C., Lam A. (2008). Plantar Fascia Thickness, a Measure of Tissue Glycation, Predicts the Development of Complications in Adolescents with Type 1 Diabetes. Diabetes Care.

[64] Benitez-Aguirre P.Z., Craig M.E., Jenkins A.J., Gallego P.H., Cusumano J., Dufin A.C., Hing S., Donaghue K.C. (2012). Plantar Fascia Thickness Is Longitudinally Associated with Retinopathy and Renal Dysfunction: A Prospective Study from Adolescence to Adulthood. J. Diabetes Sci. Technol..

[65] Ursini F., Arturi F., Nicolosi K., Ammendolia A., D’Angelo S., Russo E., Naty S., Bruno C., de Sarro G., Olivieri I. (2017). Plantar Fascia Enthesopathy Is Highly Prevalent in Diabetic Patients without Peripheral Neuropathy and Correlates with Retinopathy and Impaired Kidney Function. PLoS ONE.

[66] Khor B.Y.C., Woodburn J., Newcombe L., Barn R. (2021). Plantar Soft Tissues and Achilles Tendon Thickness and Stiffness in People with Diabetes: A Systematic Review. J. Foot Ankle Res..

[67] Ugwoke C.K., Cvetko E., Umek N. (2022). Skeletal Muscle Microvascular Dysfunction in Obesity-Related Insulin Resistance: Pathophysiological Mechanisms and Therapeutic Perspectives. Int. J. Mol. Sci..

[68] Wu H., Ballantyne C.M. (2017). Skeletal Muscle Inflammation and Insulin Resistance in Obesity. J. Clin. Investig..

[69] Money S. (2017). Pathophysiology of Trigger Points in Myofascial Pain Syndrome. J. Pain Palliat. Care Pharmacother..

[70] Kratochvil M.J., Kaber G., Demirdjian S., Cai P.C., Burgener E.B., Nagy N., Barlow G.L., Popescu M., Nicolls M.R., Ozawa M.G. (2022). Biochemical, Biophysical, and Immunological Characterization of Respiratory Secretions in Severe SARS-CoV-2 Infections. JCI Insight.

[71] Kaul A., Short W.D., Wang X., Keswani S.G. (2021). Hyaluronidases in Human Diseases. Int. J. Mol. Sci..

[72] Nagy N., Kuipers H.F., Marshall P.L., Wang E., Kaber G., Bollyky P.L. (2019). Hyaluronan in Immune Dysregulation and Autoimmune Diseases. Matrix Biol..

[73] Karousou E., Misra S., Ghatak S., Dobra K., Götte M., Vigetti D., Passi A., Karamanos N.K., Skandalis S.S. (2017). Roles and Targeting of the HAS/Hyaluronan/CD44 Molecular System in Cancer. Matrix Biol..

[74] Heldin P., Kolliopoulos C., Lin C.Y., Heldin C.H. (2020). Involvement of Hyaluronan and CD44 in Cancer and Viral Infections. Cell. Signal..

[75] Homann S., Grandoch M., Kiene L.S., Podsvyadek Y., Feldmann K., Rabausch B., Nagy N., Lehr S., Kretschmer I., Oberhuber A. (2018). Hyaluronan Synthase 3 Promotes Plaque Inflammation and Atheroprogression. Matrix Biol..

[76] Nagy N., Sunkari V.G., Kaber G., Hasbun S., Lam D.N., Speake C., Sanda S., McLaughlin T.L., Wight T.N., Long S.R. (2019). Hyaluronan Levels Are Increased Systemically in Human Type 2 but Not Type 1 Diabetes Independently of Glycemic Control. Matrix Biol..

[77] Suzuki A., Angulo P., Lymp J., Li D., Satomura S., Lindor K. (2005). Hyaluronic Acid, an Accurate Serum Marker for Severe Hepatic Fibrosis in Patients with Non-Alcoholic Fatty Liver Disease. Liver Int..

[78] Kaneda H., Hashimoto E., Yatsuji S., Tokushige K., Shiratori K. (2006). Hyaluronic Acid Levels Can Predict Severe Fibrosis and Platelet Counts Can Predict Cirrhosis in Patients with Nonalcoholic Fatty Liver Disease. J. Gastroenterol. Hepatol..

[79] Kang L., Lantier L., Kennedy A., Bonner J.S., Mayes W.H., Bracy D.P., Bookbinder L.H., Hasty A.H., Thompson C.B., Wasserman D.H. (2013). Hyaluronan Accumulates with High-Fat Feeding and Contributes to Insulin Resistance. Diabetes.

[80] Morita M., Yano S., Ishibashi Y., Nakata N., Kurioka S., Sugimoto T. (2014). Close Relationship between Serum Hyaluronan Levels and Vascular Function in Patients with Type 2 Diabetes. Biomarkers.

[81] Zhu Y., Kruglikov I.L., Akgul Y., Scherer P.E. (2019). Hyaluronan in Adipogenesis, Adipose Tissue Physiology and Systemic Metabolism. Matrix Biol..

[82] Romo M., López-Vicario C., Pérez-Romero N., Casulleras M., Martínez-Puchol A.I., Sánchez B., Flores-Costa R., Alcaraz-Quiles J., Duran-Güell M., Ibarzábal A. (2022). Small Fragments of Hyaluronan Are Increased in Individuals with Obesity and Contribute to Low-Grade Inflammation through TLR-Mediated Activation of Innate Immune Cells. Int. J. Obes..

[83] Zatterale F., Longo M., Naderi J., Raciti G.A., Desiderio A., Miele C., Beguinot F. (2020). Chronic Adipose Tissue Inflammation Linking Obesity to Insulin Resistance and Type 2 Diabetes. Front. Physiol..

[84] Fogelstrand P., Borén J. (2013). Treatment of Hyaluronan Accumulation Ameliorates High-Fat Diet-Induced Insulin Resistance in Mice. Diabetes.

[85] Hasib A., Hennayake C.K., Bracy D.P., Bugler-Lamb A.R., Lantier L., Khan F., Ashford M.L.J., McCrimmon R.J., Wasserman D.H., Kang L. (2019). CD44 Contributes to Hyaluronan-Mediated Insulin Resistance in Skeletal Muscle of High-Fat-Fed C57BL/6 Mice. Am. J. Physiol. Endocrinol. Metab..

[86] Ji E., Jung M.Y., Park J.H., Kim S., Seo C.R., Park K.W., Lee E.K., Yeom C.H., Lee S. (2014). Inhibition of Adipogenesis in 3T3-L1 Cells and Suppression of Abdominal Fat Accumulation in High-Fat Diet-Feeding C57BL/6J Mice after Downregulation of Hyaluronic Acid. Int. J. Obes..

[87] Kodama K., Horikoshi M., Toda K., Yamada S., Hara K., Irie J., Sirota M., Morgan A.A., Chen R., Ohtsu H. (2012). Expression-Based Genome-Wide Association Study Links the Receptor CD44 in Adipose Tissue with Type 2 Diabetes. Proc. Natl. Acad. Sci. USA.

[88] Avenoso A., Bruschetta G., D`Ascola A., Scuruchi M., Mandraffino G., Saitta A., Campo S., Campo G.M. (2020). Hyaluronan Fragmentation During Inflammatory Pathologies: A Signal That Empowers Tissue Damage. Mini Rev. Med. Chem..

[89] Brecht M., Mayer U., Schlosser E., Prehm P. (1986). Increased Hyaluronate Synthesis Is Required for Fibroblast Detachment and Mitosis. Biochem. J..

[90] Thomas L., Byers H.R., Vink J., Stamenkovic I. (1992). CD44H Regulates Tumor Cell Migration on Hyaluronate-Coated Substrate. J. Cell Biol..

[91] Hall C.L., Turley E.A. (1995). Hyaluronan: RHAMM Mediated Cell Locomotion and Signaling in Tumorigenesis. J. Neurooncol..

[92] Gerdin B., Hällgren R. (1997). Dynamic Role of Hyaluronan (HYA) in Connective Tissue Activation and Inflammation. J. Intern. Med..

[93] Garantziotis S., Savani R.C. (2022). Proteoglycans in Toll-like Receptor Responses and Innate Immunity. Am. J. Physiol. Cell Physiol..

[94] Mohamadzadeh M., DeGrendele H., Arizpe H., Estess P., Siegelman M. (1998). Proinflammatory Stimuli Regulate Endothelial Hyaluronan Expression and CD44/HA-Dependent Primary Adhesion. J. Clin. Investig..

[95] Kobayashi H., Terao T. (1997). Hyaluronic Acid-Specific Regulation of Cytokines by Human Uterine Fibroblasts. Am. J. Physiol..

[96] Baranova N.S., Nilebäck E., Haller F.M., Briggs D.C., Svedhem S., Day A.J., Richter R.P. (2011). The Inflammation-Associated Protein TSG-6 Cross-Links Hyaluronan via Hyaluronan-Induced TSG-6 Oligomers. J. Biol. Chem..

[97] Lui P.P.Y., Yung P.S.H. (2021). Inflammatory Mechanisms Linking Obesity and Tendinopathy. J. Orthop. Translat..

[98] MacChi M., Spezia M., Elli S., Schiaffini G., Chisari E. (2020). Obesity Increases the Risk of Tendinopathy, Tendon Tear and Rupture, and Postoperative Complications: A Systematic Review of Clinical Studies. Clin. Orthop. Relat. Res..

[99] He L., Yu T., Zhang W., Wang B., Ma Y., Li S. (2022). Causal Associations of Obesity with Achilles Tendinopathy: A Two-Sample Mendelian Randomization Study. Front. Endocrinol..

[100] Jomaa G., Kwan C.K., Fu S.C., Ling S.K.K., Chan K.M., Yung P.S.H., Rolf C. (2020). A Systematic Review of Inflammatory Cells and Markers in Human Tendinopathy. BMC Musculoskelet. Disord..

[101] Chisari E., Rehak L., Khan W.S., Maffulli N. (2019). Tendon Healing in Presence of Chronic Low-Level Inflammation: A Systematic Review. Br. Med. Bull..

[102] Wu H., Ballantyne C.M. (2020). Metabolic Inflammation and Insulin Resistance in Obesity. Circ. Res..

[103] Albano G.D., Bonanno A., Cavalieri L., Ingrassia E., di Sano C., Siena L., Riccobono L., Gagliardo R., Profita M. (2016). Effect of High, Medium, and Low Molecular Weight Hyaluronan on Inflammation and Oxidative Stress in an In Vitro Model of Human Nasal Epithelial Cells. Mediat. Inflamm..

[104] Chaudhuri O., Cooper-White J., Janmey P.A., Mooney D.J., Shenoy V.B. (2020). Effects of Extracellular Matrix Viscoelasticity on Cellular Behaviour. Nature.

[105] Grolma J.M., Weinand P., Moone D.J. (2020). Extracellular Matrix Plasticity as a Driver of Cell Spreading. Proc. Natl. Acad. Sci. USA.

[106] Bastardot F., Marques-Vidal P., Vollenweider P. (2018). Association of Body Temperature with Obesity. The CoLaus Study. Int. J. Obes..

[107] Smilek J., Jarábková S., Velcer T., Pekař M. (2019). Compositional and Temperature Effects on the Rheological Properties of Polyelectrolyte–Surfactant Hydrogels. Polymers.

[108] Matteini P., Dei L., Carretti E., Volpi N., Goti A., Pini R. (2009). Structural Behavior of Highly Concentrated Hyaluronan. Biomacromolecules.

[109] Gatej I., Popa M., Rinaudo M. (2005). Role of the PH on Hyaluronan Behavior in Aqueous Solution. Biomacromolecules.

[110] Chen C.N.J., Liao Y.H., Lin S.Y., Yu J.X., Li Z.J., Lin Y.C., Chang G.J., Lin C.H., Wong A.M.K. (2017). Diet-Induced Obesity Accelerates Blood Lactate Accumulation of Rats in Response to Incremental Exercise to Maximum. Am. J. Physiol. Regul. Integr. Comp. Physiol..

[111] Maleki A., Kjøniksen A.L., Nyström B. (2008). Effect of PH on the Behavior of Hyaluronic Acid in Dilute and Semidilute Aqueous Solutions. Macromol. Symp..

[112] Pisárčik M., Bakoš D., Čeppan M. (1995). Non-Newtonian Properties of Hyaluronic Acid Aqueous Solution. Colloids Surf. A Physicochem. Eng. Asp..

[113] Lang I.A., Llewellyn D.J., Alexander K., Melzer D. (2008). Obesity, Physical Function, and Mortality in Older Adults. J. Am. Geriatr. Soc..

[114] Vincent H.K., Vincent K.R., Lamb K.M. (2010). Obesity and Mobility Disability in the Older Adult. Obes. Rev..

[115] Hughes E.J., McDermott K., Funk M.F. (2019). Evaluation of Hyaluronan Content in Areas of Densification Compared to Adjacent Areas of Fascia. J. Bodyw. Mov. Ther..

[116] Pavan P.G., Stecco A., Stern R., Stecco C. (2014). Painful Connections: Densification versus Fibrosis of Fascia. Curr. Pain Headache Rep..

[117] Wilke J., Macchi V., de Caro R., Stecco C. (2019). Fascia Thickness, Aging and Flexibility: Is There an Association?. J. Anat..

[118] Stecco A., Meneghini A., Stern R., Stecco C., Imamura M. (2014). Ultrasonography in Myofascial Neck Pain: Randomized Clinical Trial for Diagnosis and Follow-Up. Surg. Radiol. Anat..

[119] Marcelin G., Silveira A.L.M., Martins L.B., Ferreira A.V.M., Clément K. (2019). Deciphering the Cellular Interplays Underlying Obesity-Induced Adipose Tissue Fibrosis. J. Clin. Investig..

[120] Fede C., Petrelli L., Guidolin D., Porzionato A., Pirri C., Fan C., de Caro R., Stecco C. (2021). Evidence of a New Hidden Neural Network into Deep Fasciae. Sci. Rep..

[121] Suarez-Rodriguez V., Fede C., Pirri C., Petrelli L., Loro-Ferrer J.F., Rodriguez-Ruiz D., de Caro R., Stecco C. (2022). Fascial Innervation: A Systematic Review of the Literature. Int. J. Mol. Sci..

[122] Charles D., Hudgins T., MacNaughton J., Newman E., Tan J., Wigger M. (2019). A Systematic Review of Manual Therapy Techniques, Dry Cupping and Dry Needling in the Reduction of Myofascial Pain and Myofascial Trigger Points. J. Bodyw. Mov. Ther..

[123] Barbero M., Schneebeli A., Koetsier E., Maino P. (2019). Myofascial Pain Syndrome and Trigger Points: Evaluation and Treatment in Patients with Musculoskeletal Pain. Curr. Opin. Support Palliat. Care.

[124] Galasso A., Urits I., An D., Nguyen D., Borchart M., Yazdi C., Manchikanti L., Kaye R.J., Kaye A.D., Mancuso K.F. (2020). A Comprehensive Review of the Treatment and Management of Myofascial Pain Syndrome. Curr. Pain Headache Rep..

[125] Thottungal A., Kumar P., Bhaskar A. (2019). Interventions for Myofascial Pain Syndrome in Cancer Pain: Recent Advances: Why, When, Where and How. Curr. Opin. Support Palliat. Care..

[126] Urits I., Charipova K., Gress K., Schaaf A.L., Gupta S., Kiernan H.C., Choi P.E., Jung J.W., Cornett E., Kaye A.D. (2020). Treatment and Management of Myofascial Pain Syndrome. Best Pract. Res. Clin. Anaesthesiol..

[127] Abatangelo G., Vindigni V., Avruscio G., Pandis L., Brun P. (2020). Hyaluronic Acid: Redefining Its Role. Cells.

[128] Hamidi M., Azadi A., Rafiei P. (2008). Hydrogel Nanoparticles in Drug Delivery. Adv. Drug Deliv. Rev..

[129] Serban M.A., Skardal A. (2019). Hyaluronan Chemistries for Three-Dimensional Matrix Applications. Matrix Biol..

[130] Buck D.W., Alam M., Kim J.Y.S. (2009). Injectable Fillers for Facial Rejuvenation: A Review. J. Plast. Reconstr. Aesthet. Surg..

[131] Gold M.H. (2007). Use of Hyaluronic Acid Fillers for the Treatment of the Aging Face. Clin. Interv. Aging.

[132] Sun S.F., Chou Y.J., Hsu C.W., Chen W.L. (2009). Hyaluronic Acid as a Treatment for Ankle Osteoarthritis. Curr. Rev. Musculoskelet. Med..

[133] Urits I., Hasegawa M., Orhurhu V., Peck J., Kelly A.C., Kaye R.J., Orhurhu M.S., Brinkman J., Giacomazzi S., Foster L. (2020). Minimally Invasive Treatment of Chronic Ankle Instability: A Comprehensive Review. Curr. Pain Headache Rep..

[134] Papalia R., Albo E., Russo F., Tecame A., Torre G., Sterzi S., Bressi F., Denaro V. (2017). The Use of Hyaluronic Acid in the Treatment of Ankle Osteoarthritis: A Review of the Evidence. J. Biol. Regul. Homeost. Agents.

[135] Honda H., Gotoh M., Kanazawa T., Ohzono H., Nakamura H., Ohta K., Nakamura K.I., Fukuda K., Teramura T., Hashimoto T. (2017). Hyaluronic Acid Accelerates Tendon-to-Bone Healing After Rotator Cuff Repair. Am. J. Sports Med..

[136] Osti L., Berardocco M., di Giacomo V., di Bernardo G., Oliva F., Berardi A.C. (2015). Hyaluronic Acid Increases Tendon Derived Cell Viability and Collagen Type i Expression in Vitro: Comparative Study of Four Different Hyaluronic Acid Preparations by Molecular Weight. BMC Musculoskelet. Disord..

[137] Crimaldi S., Liguori S., Tamburrino P., Moretti A., Paoletta M., Toro G., Iolascon G. (2021). The Role of Hyaluronic Acid in Sport-Related Tendinopathies: A Narrative Review. Medicina.

[138] Kogan G., Šoltés L., Stern R., Gemeiner P. (2007). Hyaluronic Acid: A Natural Biopolymer with a Broad Range of Biomedical and Industrial Applications. Biotechnol. Lett..

[139] Abate M., Schiavone C., Salini V. (2014). The Use of Hyaluronic Acid after Tendon Surgery and in Tendinopathies. Biomed. Res. Int..

[140] Kaux J.-F., Samson A., Crielaard J.-M. (2015). Hyaluronic Acid and Tendon Lesions. Muscles Ligaments Tendons J..

[141] Giordan N., Giordan N., Mazzoni G. (2017). Efficacy and Safety of Hyaluronic Acid (500-730kDa) Ultrasound-Guided Injections on Painful Tendinopathies: A Prospective, Open Label, Clinical Study. Muscles Ligaments Tendons J..

[142] Oliva F., Marsilio E., Asparago G., Frizziero A., Berardi A.C., Maffulli N. (2021). The Impact of Hyaluronic Acid on Tendon Physiology and Its Clinical Application in Tendinopathies. Cells.

[143] Fede C., Pirri C., Petrelli L., Guidolin D., Fan C., de Caro R., Stecco C. (2020). Sensitivity of the Fasciae to the Endocannabinoid System: Production of Hyaluronan-Rich Vesicles and Potential Peripheral Effects of Cannabinoids in Fascial Tissue. Int. J. Mol. Sci..

[144] Raeissadat S.A., Nouri F., Darvish M., Esmaily H., Ghazihosseini P. (2020). Ultrasound-Guided Injection of High Molecular Weight Hyaluronic Acid versus Corticosteroid in Management of Plantar Fasciitis: A 24-Week Randomized Clinical Trial. J. Pain Res..

[145] Kumai T., Muneta T., Tsuchiya A., Shiraishi M., Ishizaki Y., Sugimoto K., Samoto N., Isomoto S., Tanaka Y., Takakura Y. (2014). The Short-Term Effect after a Single Injection of High-Molecular-Weight Hyaluronic Acid in Patients with Enthesopathies (Lateral Epicondylitis, Patellar Tendinopathy, Insertional Achilles Tendinopathy, and Plantar Fasciitis): A Preliminary Study. J. Orthop. Sci..

[146] Ferreira G.F., Sevilla D., Oliveira C.N., Junior L.C.N., Arliani G.G., Oliveira V.O., Filho M.V.P. (2021). Comparison of the Effect of Hyaluronic Acid Injection versus Extracorporeal Shockwave Therapy on Chronic Plantar Fasciitis: Protocol for a Randomized Controlled Trial. PLoS ONE.

[147] Pozo M.A., Balazs E.A., Belmonte C. (1997). Reduction of Sensory Responses to Passive Movements of Inflamed Knee Joints by Hylan, a Hyaluronan Derivative. Exp. Brain Res..

[148] Moreland L.W. (2003). Intra-Articular Hyaluronan (Hyaluronic Acid) and Hylans for the Treatment of Osteoarthritis: Mechanisms of Action. Arthritis Res. Ther..

[149] Eymard F., Chevalier X., Conrozier T. (2017). Obesity and Radiological Severity Are Associated with Viscosupplementation Failure in Patients with Knee Osteoarthritis. J. Orthop. Res..

[150] Conrozier T., Eymard F., Chouk M., Chevalier X. (2019). Impact of Obesity, Structural Severity and Their Combination on the Efficacy of Viscosupplementation in Patients with Knee Osteoarthritis. BMC Musculoskelet. Disord..

[151] Coombes B.K., Bisset L., Vicenzino B. (2010). Efficacy and Safety of Corticosteroid Injections and Other Injections for Management of Tendinopathy: A Systematic Review of Randomised Controlled Trials. Lancet.

[152] Kumai T., Samoto N., Hasegawa A., Noguchi H., Shiranita A., Shiraishi M., Ikeda S., Sugimoto K., Tanaka Y., Takakura Y. (2018). Short-Term Efficacy and Safety of Hyaluronic Acid Injection for Plantar Fasciopathy. Knee Surg. Sports Traumatol. Arthrosc..

[153] Lynen N., de Vroey T., Spiegel I., van Ongeval F., Hendrickx N.J., Stassijns G. (2017). Comparison of Peritendinous Hyaluronan Injections Versus Extracorporeal Shock Wave Therapy in the Treatment of Painful Achilles’ Tendinopathy: A Randomized Clinical Efficacy and Safety Study. Arch. Phys. Med. Rehabil..

[154] Mohebbi R., Rezasoltani Z., Mir M., Mohebbi M., Vatandoost S., Esmaily H. (2021). High- Versus Low-Molecular-Weight Hyaluronic Acid for the Treatment of Rotator Cuff Tendinopathy: A Triple-Blind Randomized Comparative Trial. Ann. Pharmacother..

[155] Bahrami M.H., Raeissadat S.A., Cheraghi M., Rahimi-Dehgolan S., Ebrahimpour A. (2020). Efficacy of Single High-Molecular-Weight versus Triple Low-Molecular-Weight Hyaluronic Acid Intra-Articular Injection among Knee Osteoarthritis Patients. BMC Musculoskelet. Disord..

[156] Petrey A.C., de la Motte C.A. (2014). Hyaluronan, a Crucial Regulator of Inflammation. Front. Immunol..

[157] Jiang D., Liang J., Noble P.W. (2011). Hyaluronan as an Immune Regulator in Human Diseases. Physiol. Rev..

[158] Chin L., Calabro A., Walker E., Derwin K.A. (2012). Mechanical Properties of Tyramine Substituted-Hyaluronan Enriched Fascia Extracellular Matrix. J. Biomed. Mater. Res. A.

[159] Marinho A., Nunes C., Reis S. (2021). Hyaluronic Acid: A Key Ingredient in the Therapy of Inflammation. Biomolecules.

[160] Fede C., Albertin G., Petrelli L., Sfriso M.M., Biz C., de Caro R., Stecco C. (2016). Expression of the Endocannabinoid Receptors in Human Fascial Tissue. Eur. J. Histochem..

[161] Ghasemi M., Mosaffa F., Hoseini B., Behnaz F. (2020). Comparison of the Effect of Bicarbonate, Hyaluronidase, and Lidocaine Injection on Myofascial Pain Syndrome. Anesth. Pain Med..

[162] Raghavan P., Lu Y., Mirchandani M., Stecco A. (2016). Human Recombinant Hyaluronidase Injections for Upper Limb Muscle Stiffness in Individuals with Cerebral Injury: A Case Series. eBioMedicine.

